# Supplementary material for: Procr-expressing progenitor cells are responsible for murine ovulatory rupture repair of ovarian surface epithelium
Source: Nat Commun. 2019 Oct 31;10:4966. doi: 10.1038/s41467-019-12935-7 (PMC6823351; doi:10.1038/s41467-019-12935-7)
Supplement: Supplementary file 1 — Supplementary Information [file 41467_2019_12935_MOESM1_ESM.pdf]

Supplementary Information

Procr-expressing progenitor cells are responsible for murine ovulatory rupture repair of ovarian surface epithelium

Wang et al.

Wang et al., Supplementary figure 1, Generation of *Procr-rtTA* knock-in allele

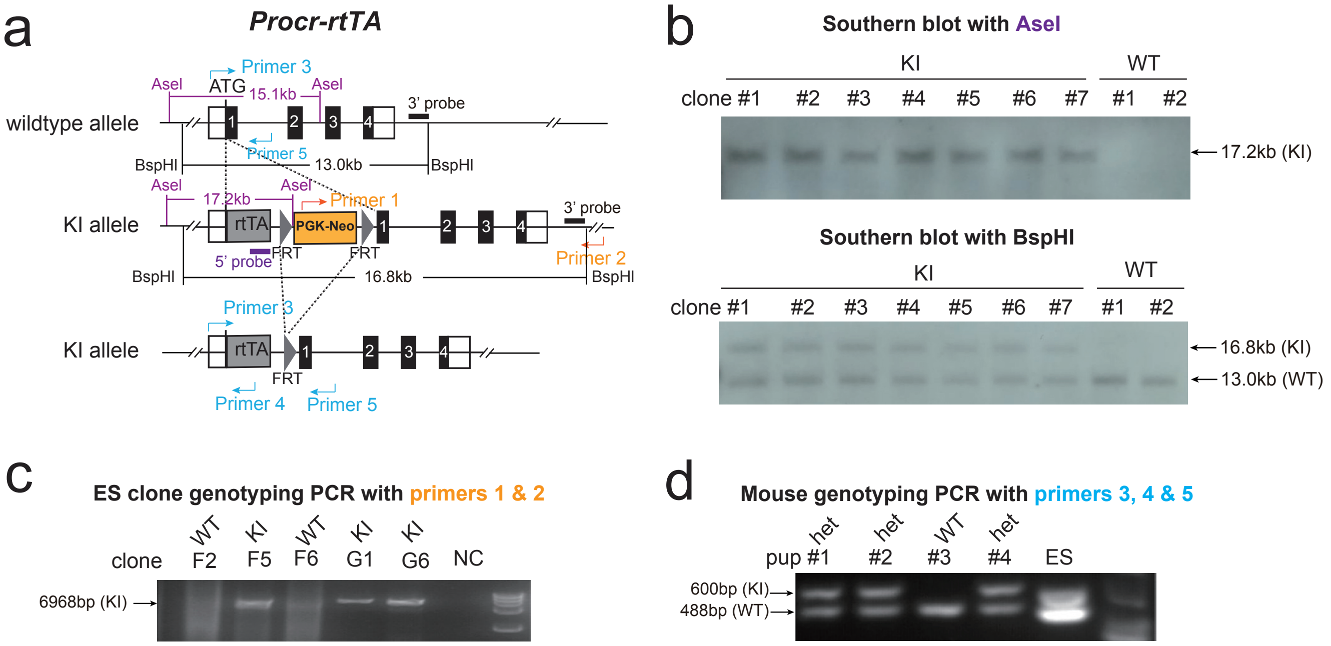

a, Targeting strategy to generate the *Procr-rtTA* knock-in mouse. Designs of southern blot probe (magenta or black), ES clone genotyping primers (orange) and mouse genotyping primers (turquoise) are as indicated.
b, Southern blot analysis with a 5' external probe of AseI-digested DNA from mouse embryonic stem cells, showing a 17.2 kb band (top), and with a 3' external probe of BspHI-digested DNA from mouse embryonic stem cells, showing a 16.8 kb band in addition to the 13.0 kb WT band (bottom) in clones that have undergone homologous recombination at the Procr locus.
c, ES clone genotyping PCR indicating clone F5, G1, G6 are with successful knock-in (KI). One of three similar experiments is shown. NC, negative control with no DNA input.
d, Genotyping PCR indicating pub #1, 2, 4 are heterozygotes, #3 is wildtype. A positive ES clone was used as positive control. One of three similar experiments is shown.

Wang et al, Supplementary figure 2. Establishing FACS analysis methods for mouse OSE cells

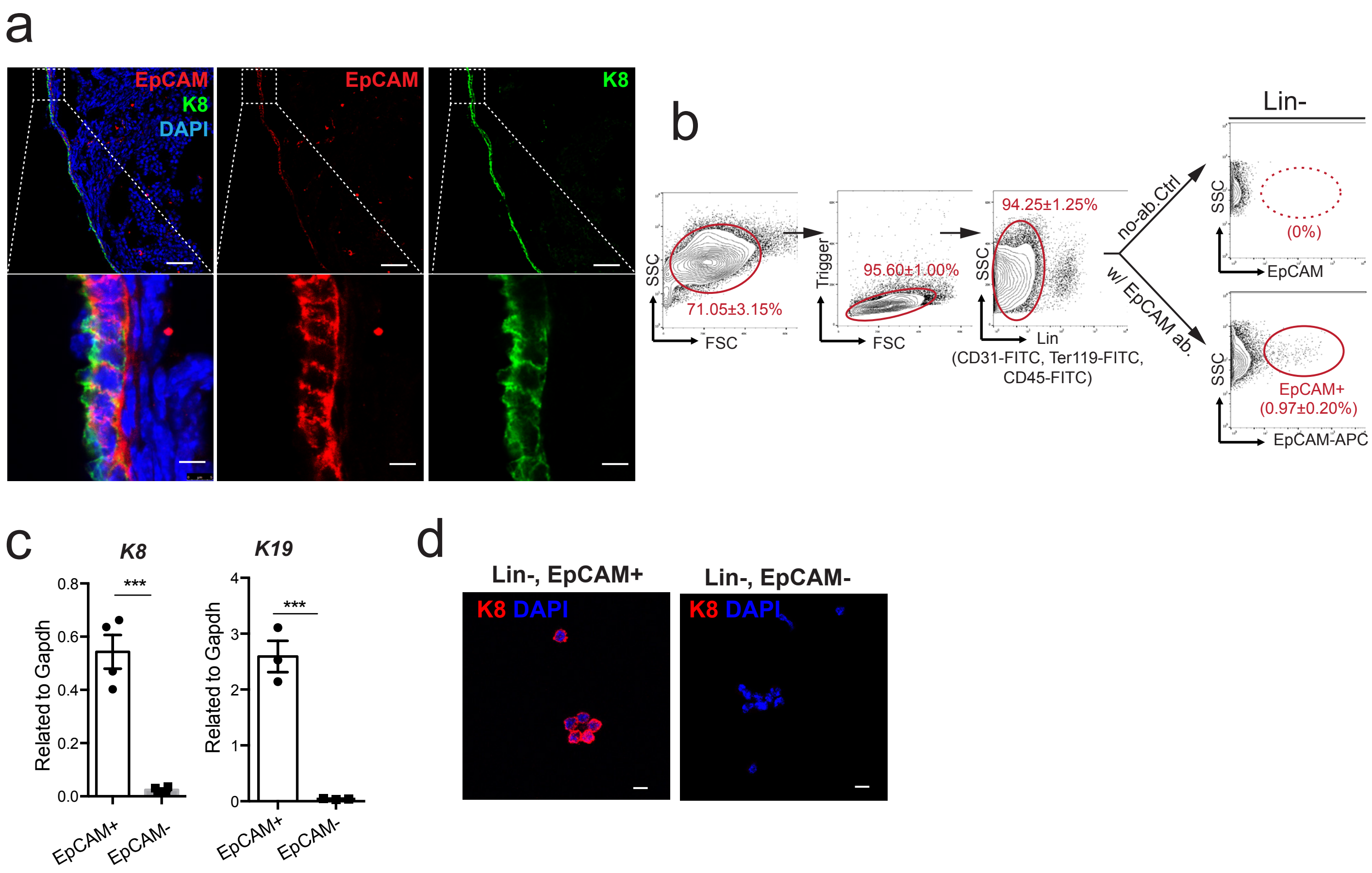

a, Immunohistochemistry of EpCAM and K8 indicating the co-expression of EpCAM and K8 in the adult mouse ovarian surface epithelium. n=3 mice. Scale bars, 50μm in large view and 5μm in zoom-in view.

b, FACS plot for OSE cells in the ovary. Endothelial and blood lineage (Lin+) cells were excluded by CD31, CD45, Ter119 antibodies. EpCAM+ cells occupied about 1% of all ovarian cells. One of three similar experiments is shown.

c, qPCR analyses indicating that isolated Lin-, EpCAM+ cells have drastically higher K8 and K19 expression compared to Lin-, EpCAM- cells, validating the OSE identity of Lin-, EpCAM+ cells. Data are pooled from 3 independent experiments and displayed as mean±s.e.m. ***P<0.001.

d, Immunohistochemistry confirming the positive K8 expression in isolated and cytospun Lin-, EpCAM+ cells, while Lin-, EpCAM- cells are K8-negative. Scale bars, 10μm. n=3 mice.

Wang et al., Supplementary figure 3. Procr+ cells contribute to OSE homeostasis in adult

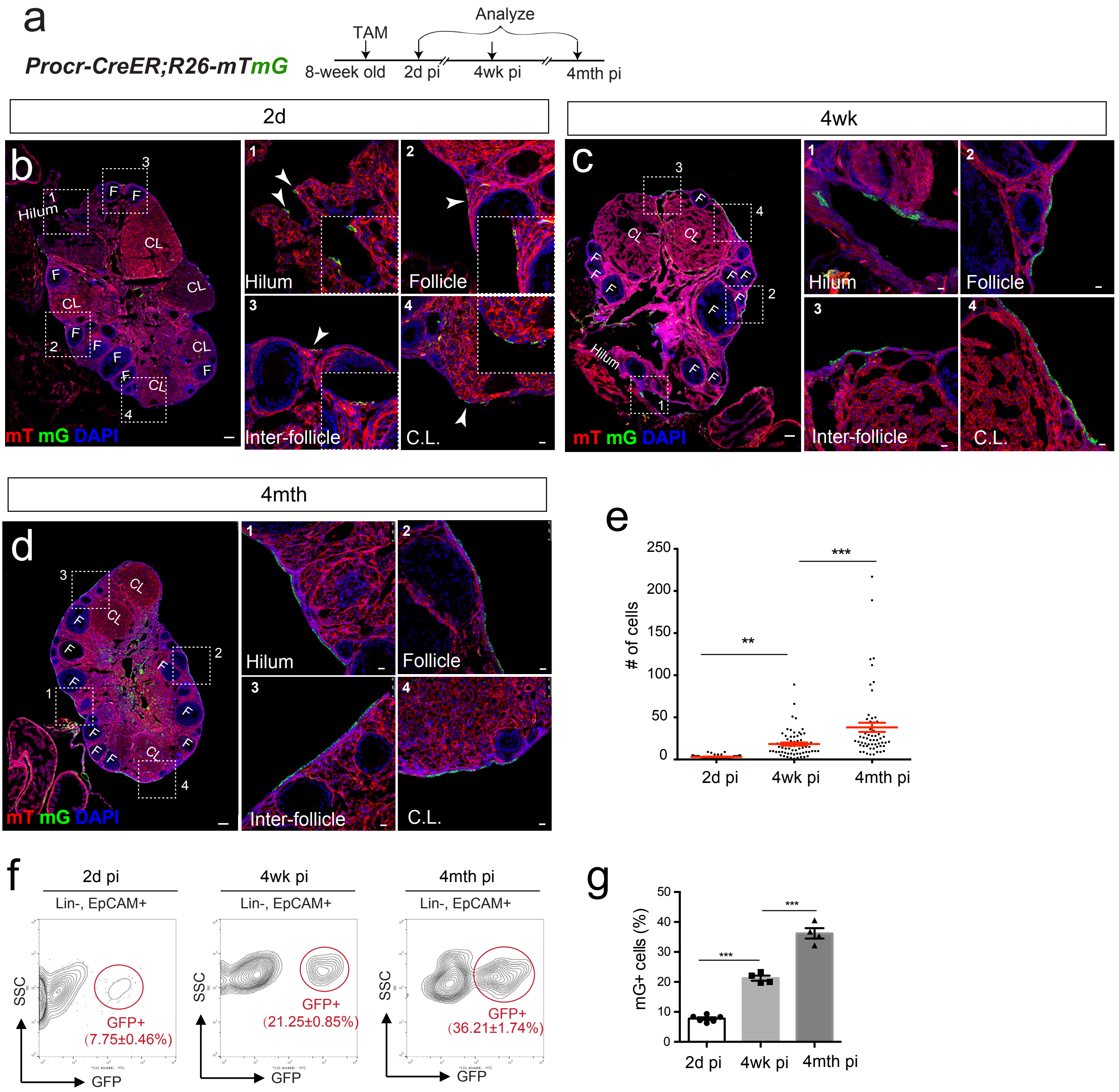

a, Illustration of lineage tracing strategy in 8-week old adult *Procr-CreER;R26-mTmG* mice. The labelled progeny were analysed after short-term (2 days) and long-term traced periods (4 weeks, 4 months).

b, Section images showing that at 2 days pi, individual GFP+ cells are seen across all regions of OSE. Scale bars, 100µm in full ovary view and 20µm in zoom in view. n=3 mice.

c-d, Section images showing GFP+ clones at 4 weeks (c) and 4 months (d) across OSE. Scale bars, 100µm in full ovary view and 20µm in zoom in view. n=3 mice for each time point.

e, Quantification of GFP+ cell numbers (per 0.04mm^2 dimension) showing the continuous expansion of GFP+ cells at 2 days, 4 weeks, 4 months pi. n=3 mice for each tracing time point. ***P<0.001.

f-g, Quantification of GFP+ cell (with Lin-, EpCAM+ OSE compartment) percentages by FACS (f) showing the continuous expansion of GFP+ cells at 2 days, 4 weeks, 4 months pi (g). n=3 mice for each tracing time point. ***P<0.001.

Wang et al., Supplementary figure 4. Procr+ OSE cells lineage tracing in pubertal mice

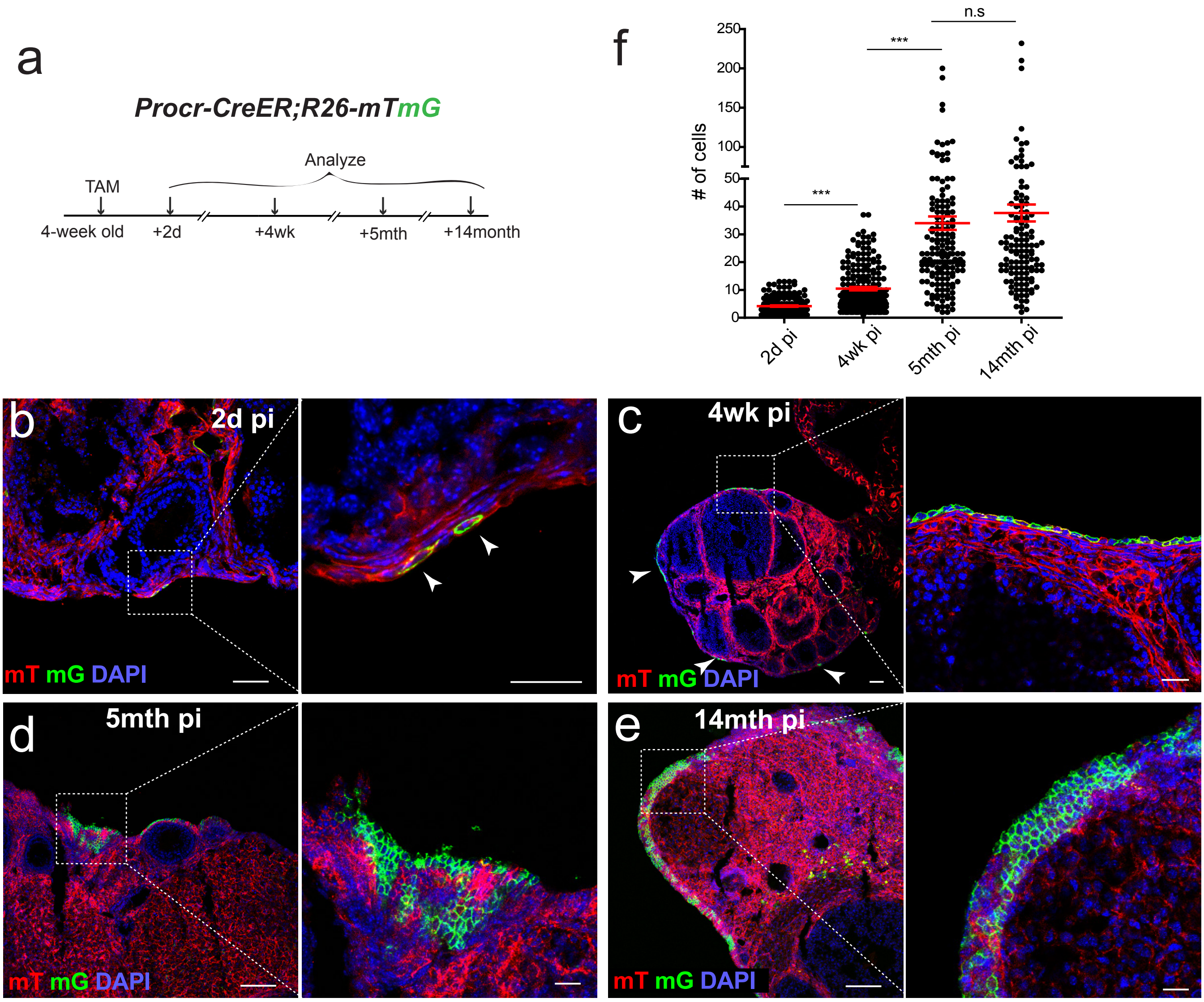

a, Illustration of lineage tracing strategy in 4-week old pubertal *Procr-CreER;R26-mTmG* mice. The labelled progeny were analysed after short-term (2 days) and long-term traced periods (4 weeks, 5 months, 14 months).

b-e, Section images showing GFP+ clones at 2 days (b), 4 weeks (c), 5 months (d), 14 months (e). Scale bars, 100μm and 20μm for zoom in. n=3 mice for each time point.

f, Quantification of GFP+ cell numbers (per $0.04mm^2$ dimension) showing the expansion of GFP+ cells along time. Data were pooled from at least 3 mice for each time point. ***P<0.001. n.s, not significant.

Supplementary figure 5. Chase the division of Procr+ cells during OSE rupture and repair

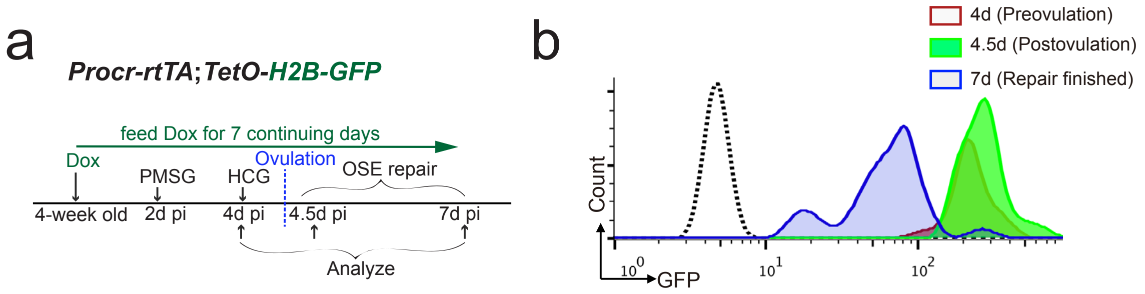

a, Illustration of the FACS analysis strategy for H2B-GFP+ cells. *Procr-rtTA;TetO-H2B-GFP* mice were fed with Doxycycline (Dox) for 7 continuing days. At 2 days, PMSG was injected, followed by HCG injection at 4 days. The analyzed time points are as indicated.

b, FACS analysis of Lin-, EpCAM+, GFP+ cells at 4d, 4.5d and 7d as indicated. n=3 mice for each time point. GFP- signal is indicated in dash line.

Wang et al., Supplementary figure 6, Tracking Procr+ cells and their progeny using H2B-GFP retention

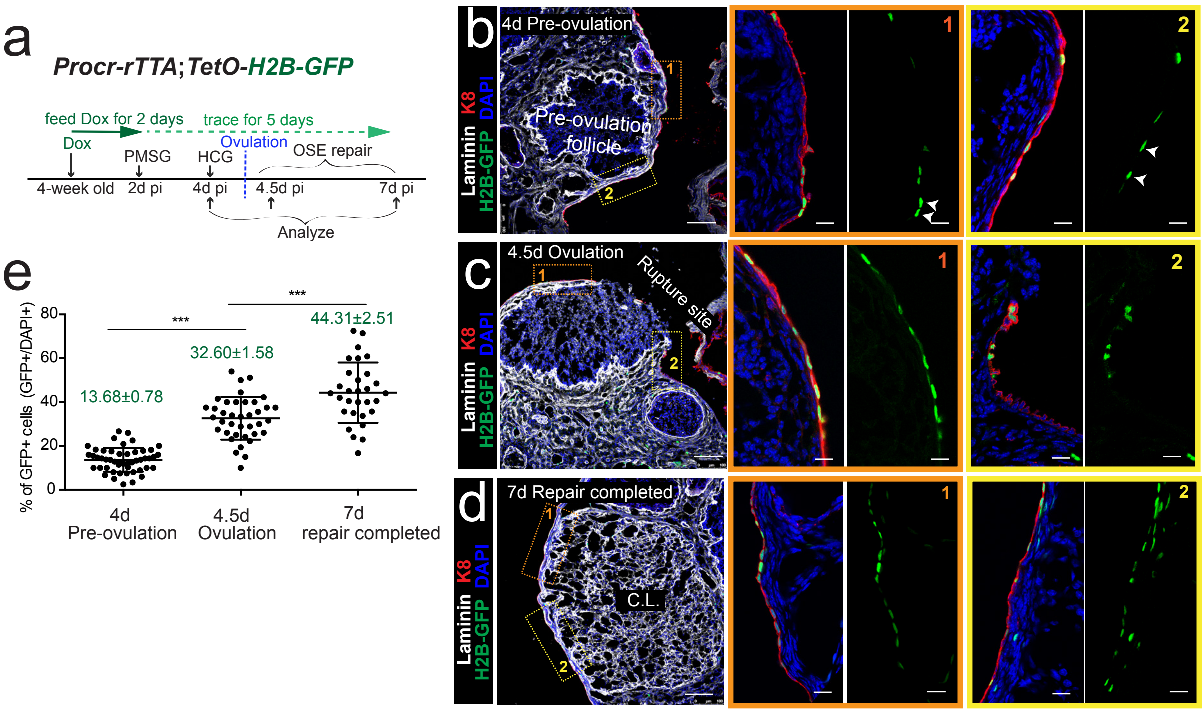

a,*Procr-rtTA;TetO-H2B-GFP* adult mice were fed with Doxycycline (Dox) for 2 days, followed by a 5-day chase. At 2 days, PMSG was injected, followed by HCG injection at 4 days. The analyzing time points are as indicated.
b, At 4 days after Dox feeding (pre-ovulation), confocal images showing a few GFP+ cells in the OSE close to pre-ovulation follicle. Scale bars, 100μm.
c, At 4.5d pi (ovulation), confocal images showing increasing numbers of GFP+ cells in the OSE on both edges of rupture follicle. Scale bars, 100μm.
d, At 7d pi (repair completed), confocal images showing abundant GFP+ cells in the OSE covering the newly formed corpus luteum. Scale bars, 100μm. n=3 mice.
e, Percentages of total GFP+ cells in the OSE covering pre-ovulation follicle, ovulating follicle, and corpus luteum were quantified, showing that GFP+ cells increase over time. n=3 mice in each time point. Data are presented as mean±s.e.m. ***P<0.001.

Wang et al., Supplementary figure 7. Procr+ cells are essential for OSE homeostasis

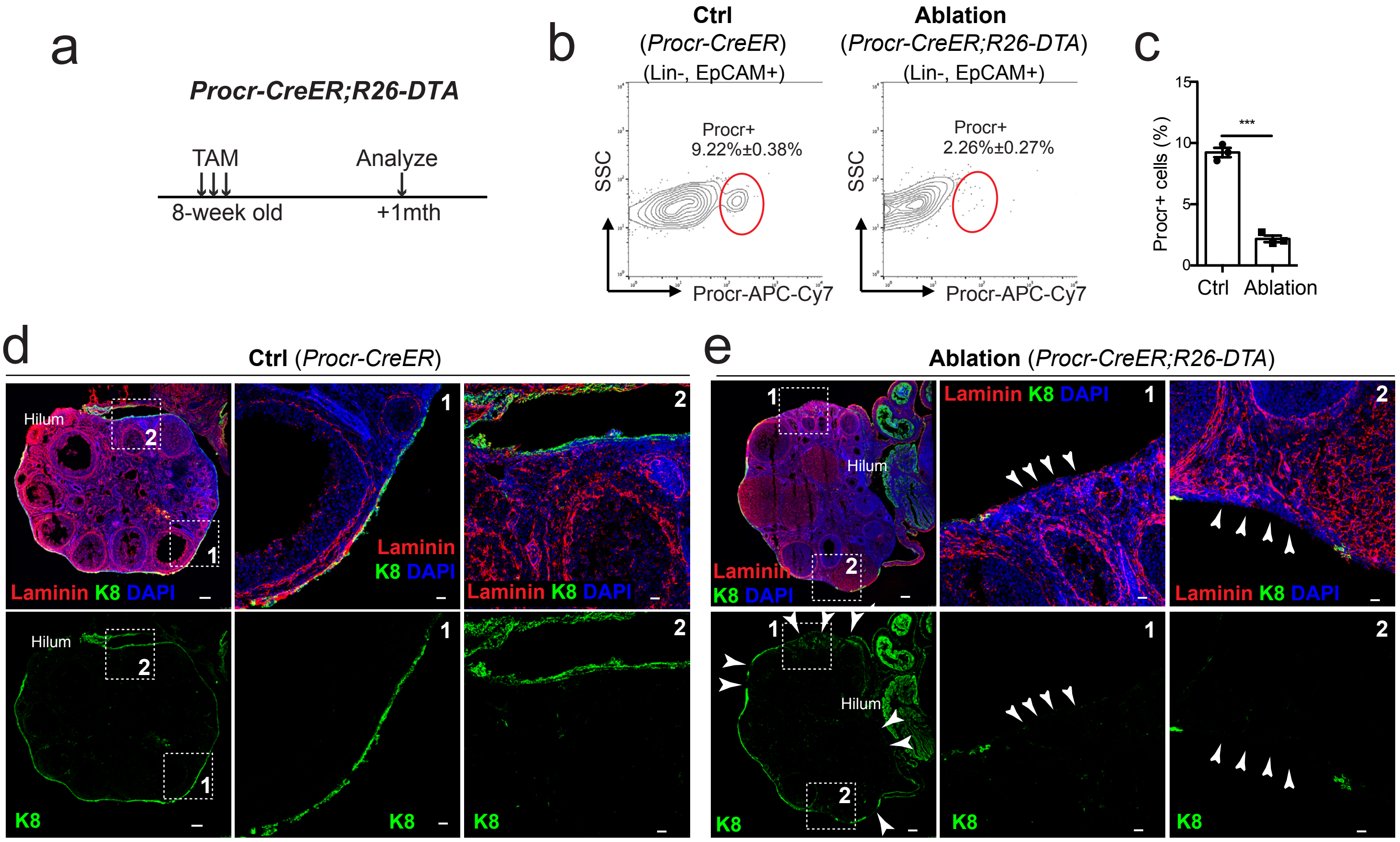

a, Illustration of the targeted cell ablation strategy using *Procr-CreER;R26-DTA* mice.
b-c, FACS analysis (b) and quantification (c) indicating the ablation efficiency of Procr+ OSE cells (Lin-, EpCAM+, Procr+). Data are displayed as mean±s.e.m. ***P<0.001. n=3 mice in each group.
d-e, Section images showing intact OSE by K8 expression in control *Procr-CreER* mice (d) and loss of OSE in *Procr-CreERT2;R26-DTA* mice (e). Arrowheads indicating the areas lack of K8 staining (e). Scale bars, 100μm and 20μm for zoom in. n=3 mice in each group.
