# Supplementary material for: Procr-expressing progenitor cells are responsible for murine ovulatory rupture repair of ovarian surface epithelium
Source: Nat Commun. 2019 Oct 31;10:4966. doi: 10.1038/s41467-019-12935-7 (PMC6823351; doi:10.1038/s41467-019-12935-7)
Supplement: Supplementary file 3 — Reporting Summary [file 41467_2019_12935_MOESM3_ESM.pdf]

nature research

Corresponding author(s): Yi Arial Zeng

Last updated by author(s): Oct 1, 2019

Reporting Summary

Nature Research wishes to improve the reproducibility of the work that we publish. This form provides structure for consistency and transparency in reporting. For further information on Nature Research policies, see Authors & Referees and the Editorial Policy Checklist .

Statistics

For all statistical analyses, confirm that the following items are present in the figure legend, table legend, main text, or Methods section.

n/a	Confirmed	
☐	☒	The exact sample size (*n*) for each experimental group/condition, given as a discrete number and unit of measurement
☐	☒	A statement on whether measurements were taken from distinct samples or whether the same sample was measured repeatedly
☐	☒	The statistical test(s) used AND whether they are one- or two-sided<br>*Only common tests should be described solely by name; describe more complex techniques in the Methods section.*
☒	☐	A description of all covariates tested
☒	☐	A description of any assumptions or corrections, such as tests of normality and adjustment for multiple comparisons
☐	☒	A full description of the statistical parameters including central tendency (e.g. means) or other basic estimates (e.g. regression coefficient) AND variation (e.g. standard deviation) or associated estimates of uncertainty (e.g. confidence intervals)
☒	☐	For null hypothesis testing, the test statistic (e.g. *F*, *t*, *r*) with confidence intervals, effect sizes, degrees of freedom and *P* value noted<br>*Give P values as exact values whenever suitable.*
☒	☐	For Bayesian analysis, information on the choice of priors and Markov chain Monte Carlo settings
☒	☐	For hierarchical and complex designs, identification of the appropriate level for tests and full reporting of outcomes
☒	☐	Estimates of effect sizes (e.g. Cohen's *d*, Pearson's *r*), indicating how they were calculated

Our web collection on statistics for biologists contains articles on many of the points above.

Software and code

Policy information about availability of computer code

Data collection	The FACS data were collected by BD FACS Jazz equipment.<br>The confocal section images were collected by Leica SP8 confocal detection system fitted on a Leica DMI6000 microscope.<br>The ovary whole mount images were collected by Leica TCS SP8 WLL.
Data analysis	The FACS data were analyzed by FlowJo (vX.0.7)<br>For bar charts, student's t-test or one-way ANOVA was performed and the p-value was calculated by GraphPad PRISM 6.

For manuscripts utilizing custom algorithms or software that are central to the research but not yet described in published literature, software must be made available to editors/reviewers. We strongly encourage code deposition in a community repository (e.g. GitHub). See the Nature Research guidelines for submitting code & software for further information.

Data

Policy information about availability of data

All manuscripts must include a data availability statement. This statement should provide the following information, where applicable:
- Accession codes, unique identifiers, or web links for publicly available datasets
- A list of figures that have associated raw data
- A description of any restrictions on data availability

All data are available in this paper

Field-specific reporting

Please select the one below that is the best fit for your research. If you are not sure, read the appropriate sections before making your selection.

Life sciences study design

All studies must disclose on these points even when the disclosure is negative.

Sample size	Sample sizes for mouse experiments were determined based on previous experience, and reference to existing literature
Data exclusions	No data were excluded from the analysis
Replication	The reproducibility for all FACS data and immunostains was confirmed by at least three independent experiments
Randomization	Littermate mice were assigned into groups on the basis of genotype
Blinding	Blinding was not used. Measurements and data reported were quantitative and did not require subjective judgement or interpretation from the investigators.

Reporting for specific materials, systems and methods

We require information from authors about some types of materials, experimental systems and methods used in many studies. Here, indicate whether each material, system or method listed is relevant to your study. If you are not sure if a list item applies to your research, read the appropriate section before selecting a response.

Materials & experimental systems

n/a	Involved in the study	
☐	☒	Antibodies
☒	☐	Eukaryotic cell lines
☒	☐	Palaeontology
☐	☒	Animals and other organisms
☒	☐	Human research participants
☒	☐	Clinical data

Methods

n/a	Involved in the study	
☒	☐	ChIP-seq
☐	☒	Flow cytometry
☒	☐	MRI-based neuroimaging

Antibodies

Antibodies used

Antibodies used for FACS:
Anti-mouse CD31-FITC (BD, clone MEC 13.3, catalogue #553372);
Anti-mouse CD45-FITC (BD, clone 30-F11, catalogue #553080);
Anti-mouse TER119-FITC (BD, clone TER119, catalogue #557915);
Anti-mouse CD31-Biotin (BD, clone MEC 13.3, catalogue #553371);
Anti-mouse CD45-Biotin (BD, clone 30-F11, catalogue #553078);
Anti-mouse Ter119-Biotin (BD, clone TER119, catalogue #553672);
Anti-mouse Procr-Biotin (eBioscience,clone 1560, catalogue # 13-2012-82); ;
Anti-mouse EpCAM-APC (eBioscience, clone G8.8, catalogue #17-5791).
FITC conjugated Sca1 (eBioscience, clone # D7, catalogue #11-5981-82)
Streptavidin-APC-Cy7 (BioLegend, catalogue #405208)
antibodies used for immunostaining:
Rabbit anti-CK19 antibody (Abbomax, polyclonal, catalogue #602-670);
Rat anti-K8 (Developmental Hybridoma Bank, catalogue #TROMA-I)
Rat anti-EpCAM (eBioscience, clone G8.8, catalogue 17-5791-82)
Rabbit anti-Laminin (Sigma, catalogue #L9393)

Validation

Antibodies validated for FACS in mouse samples:
Anti-mouse CD31-FITC, Anti-mouse CD45-FITC, Anti-mouse TER119-FITC, Anti-mouse CD31-Biotin, Anti-mouse CD45-Biotin and Anti-mouse Ter119-Biotin were validated by manufacturer websites and previous studies including Wang et al., Nature 2015; Zeng et al., Cell stem cell 2010; Shackleton et al., Nature 2006.
Anti-mouse Procr-Biotin and streptavidin-APC-Cy7 were validated by manufacturer websites.
Anti-mouse EpCAM-APC was validated by manufacturer websites and previous studies including Huch et al., The EMBO Journal, 2013.
Anti-mouse Sca1-FITC was validated by manufacturer websites and previous studies including Xu et al. Nature communications 2018
Antibodies validated for immunostaining in mouse samples:
Rabbit anti-CK19 antibody was validated by manufacturer websites and previous studies including Goldman et al., Cell stem cell 2013.
Rat anti-CK8 antibody was validated by manufacturer websites and previous studies including Wang et al., Nature 2015

Rat anti-mouse EpCAM was validated by manufacturer websites and previous studies including Huch et al., The EMBO Journal, 2013.
Rabbit anti-mouse Laminin was validated by manufactuerr websites and previous studies including Sojka et al., Development 2014.

Animals and other organisms

Policy information about studies involving animals; ARRIVE guidelines recommended for reporting animal research

Laboratory animals	Mice, C57B6 or ICR, female, 4weeks and 8-12weeks.
Wild animals	The study do not involve wild animals.
Field-collected samples	The study do not involve samples collected from the field.
Ethics oversight	Animal Care and Use Committee of Shanghai Institute of Biochemistry and Cell Biology, Chinese Academy of Sciences.

Note that full information on the approval of the study protocol must also be provided in the manuscript.

Flow Cytometry

Plots

Confirm that:

- [x] The axis labels state the marker and fluorochrome used (e.g. CD4-FITC).
- [x] The axis scales are clearly visible. Include numbers along axes only for bottom left plot of group (a 'group' is an analysis of identical markers).
- [x] All plots are contour plots with outliers or pseudocolor plots.
- [x] A numerical value for number of cells or percentage (with statistics) is provided.

Methodology

Sample preparation	Ovaries from 7 to 12 weeks old female mice were isolated, and the bursa and oviduct were cleared out under dissect microscope with tined tweezers. The minced ovarian pieces were placed in 10ml digest buffer (RPMI 1640 (Thermo Fisher, catalogue #12633-012) with 5% fetal bovine serum (FBS, Hyclone), 1% penicillin-streptomycin (Thermo Fisher, catalogue #15140122), 25mM HEPES and 300U/ml collagenase IV (Worthington, catalogue # LS004189)) and digested at 37 °C, 100 rpm for 1hour. After lysis of the red blood cells with buffer (Sigma, catalogue #R7757) in room temperature for 5 min and centrifugation at 1000rpm for 5 min, single cells were obtained with 0.05% trypsin-EDTA treatment (Thermo Fisher, catalogue # 25200056) at 37°C for 5 min, followed by 0.1mg/ml DNaseI (Sigma, catalogue #D4263) incubation at 37°C for 5 min with gently pipetting before filtering through 70 μm cell strainers
Instrument	FACS Jazz
Software	Raw FACS data were collected using the software of FCASJazz (Becton Dickinson), FlowJo (version X.0.7) was used for data analysis.
Cell population abundance	The purity of sorted population was routinely checked by immunofluorescence staining and ensured to be more than 90%.
Gating strategy	Gating strategy examples are showed in supplementary fig 2. We first gated all preliminary events with FSC and SSC, adhesive cells are excluded by trigger pulse width, then all single cells were gated by Lin, EpCAM and Procr mainly based on the negative unstained controls and the contour or pseudocolor plot patterns.

- [x] Tick this box to confirm that a figure exemplifying the gating strategy is provided in the Supplementary Information.
